# Temporal patterns in biosecurity-related regulation before and after *Loper Bright*

**DOI:** 10.3389/fbioe.2026.1838260

**Published:** 2026-07-13

**Authors:** Daniel Irowa-Omoregie, Kavya Sanghavi, Shane Reader, Adejare Atanda

**Affiliations:** 1 Johns Hopkins Bloomberg School of Public Health, Baltimore, MD, United States; 2 MedStar Health Research Institute, Columbia, MD, United States; 3 American Association for the Advancement of Science, Washington, DC, United States; 4 RAND Corporation, Arlington, VA, United States

**Keywords:** biosecurity, Chevron deference, judicial decisions, *Loper Bright*, regulatory action

## Abstract

**Overview:**

The 2024 Supreme Court decision in *Loper Bright Enterprises v. Raimondo* ended *Chevron* deference, reshaping the relationship between federal agencies and the judiciary. This study examines the impact of this legal shift on U.S. biosecurity governance, specifically focusing on the regulation of synthetic nucleic acid technologies.

**Methods:**

We employed a multi-period observational design to analyze regulatory issuance counts across 26 federal agencies using six sentinel search terms: biosecurity, biotechnology, bioeconomy, synthetic nucleic acid, gene synthesis, and nucleic acid screening. Primary data were drawn from Regulations.gov website searches; a parallel Federal Register API dataset was constructed to enable rolling-window placebo analysis, administration transition analysis partitioned at 20 January 2025, and a matched certiorari-period baseline reconstruction. Analyses were designed to assess whether observed patterns are consistent with a Loper Bright effect, while accounting for the concurrent administration transition and baseline variability in regulatory output.

**Results:**

Total biosecurity-relevant regulatory activity declined by 21.5% in the year following *Loper Bright*, even as overall federal regulatory output increased by 2.0%. This decline was not fully explained by administration policy preferences, as biosecurity activity increased by 12.2% from the Trump to Biden administrations, though the concurrent transition to a new administration in January 2025 represents a confound the observational design cannot fully disentangle. Domains with low statutory clarity, such as gene synthesis (−87.5%), showed the most severe contractions, more extreme than 99% of historical year-over-year fluctuations, while those with clear mandates, like the bioeconomy (+200%), remained resilient.

**Discussion:**

The findings suggest that statutory clarity is strongly correlated with regulatory resilience. The post-*Loper* landscape has created significant governance gaps in novel technical domains where federal departments or agencies previously relied on interpretive authority. Legislative intervention may be required to provide the “clear statutory authority” necessary to maintain a robust national biosecurity posture.

## 1 Introduction

The Supreme Court’s 2024 decision in *Loper Bright Enterprises v. Raimondo* (Loper Bright Enterprises v. Raimondo, 2024) marks one of the most consequential administrative law rulings in decades. For forty years, the doctrine known as the Chevron Deference led courts to defer to federal agencies when statutory language was unclear, recognizing that federal departments and agencies possess technical expertise and political accountability. The majority opinion in *Loper Bright* held that courts, not agencies, are the appropriate interpreters of statutory ambiguity, and that Congress must provide the legal basis for regulatory choices rather than leaving agencies to resolve ambiguity through their own interpretive authority. This recalibration has significant ramifications in biosecurity, especially in the governance of dual-use technology. In this article, we examine synthetic nucleic acid governance within the post-*Loper Brig*ht landscape, analyzing emerging trends in the absence of Chevron deference to agency statutory interpretation.

### 1.1 Chevron deference

In Chevron U.S.A. et al. (1984), the Supreme Court established what became the central doctrine of modern administrative law. The case concerned an Environmental Protection Agency (EPA) interpretation of the Clean Air Act defining an entire industrial facility as a single “stationary source” of pollution, instead of treating each pollution-emitting device as a separate source. The Court articulated a two-step framework for federal courts to use while reviewing ambiguous statutory interpretation. First, courts ask “whether Congress has directly spoken to the precise question at issue.” Second, “if the statute is silent or ambiguous with respect to the specific issue, the question for the court is whether the agency’s answer is based on a permissible construction of the statute.”

The Court opined that agencies, in contrast to judges, are staffed with technical subject-matter experts and are politically supervised by the executive branch, thus establishing the Chevron Deference. Over time, *Chevron* became one of the most cited decisions in Supreme Court history, referenced in more than 18,000 federal court decisions (Barnett and Walker, 2024). Importantly, many domains central to the modern bioeconomy, including FDA oversight, environmental biosafety regulation, and public health emergency response, were influenced by judicial deference to agency expertise.

While *Chevron* was not absolute and applied only when statutes were genuinely ambiguous, it provided agencies with a zone of autonomous discretion to apply their expertise. This interpretive space allowed agencies to adapt frameworks to evolving technologies. In scientific domains characterized by rapid innovation, this flexibility became a core feature of federal regulatory capacity.

### 1.2 Loper Bright v. Raimondo

In Loper Bright Enterprises v. Raimondo (2024), the Supreme Court formally overruled *Chevron*. The case arose when commercial fishing companies challenged a National Marine Fisheries Service (NMFS) rule requiring fisheries to pay for onboard federal monitors. The relevant statute authorized monitoring programs but did not specify who must bear the cost. Under *Chevron*, lower courts upheld NMFS’s interpretation as a permissible construction of an ambiguous statute. However, in a 6–3 opinion authored by Chief Justice John Roberts, the Court explicitly determined that *Chevron* was incompatible with the Administrative Procedure Act (APA; 1946), which states that “the reviewing court shall decide all relevant questions of law, interpret constitutional and statutory provisions, and determine the meaning or applicability of the terms of an agency action.” Courts are now directed to “exercise their independent judgment in deciding whether an agency has acted within its statutory authority, as the APA requires” and “may not defer to an agency interpretation of the law simply because a statute is ambiguous.” Additionally, in the same Term, the Court further reshaped administrative litigation in Corner Post and Inc. v. Board of Governors of the Federal Reserve System (2024), holding that the six-year statute of limitations under the APA accrues when a plaintiff is injured, and not when a rule is issued, thereby expanding opportunities to challenge longstanding regulations.

It is important to note that *Loper Bright* does not eliminate all forms of deference. The Court emphasized that the APA “does mandate that judicial review of agency policymaking and factfinding be deferential.” Courts may still give respectful weight to agency reasoning based on the precedent of Skidmore v. Swift and Co (1944) based on which courts are to weigh various factors that include “the thoroughness evident in (the agency’s) consideration, the validity of its reasoning, its consistency with earlier and later pronouncements, and all those factors which give it the power to persuade.”

### 1.3 Loper Bright in context

The most immediate anticipated consequence of *Loper Bright* is that regulations previously sustained under Chevron deference may now be more vulnerable to judicial challenge. Under Chevron, agencies could interpret ambiguous statutory provisions in light of evolving technical realities. In the post-*Loper Bright* era, those interpretations may face more aggressive judicial scrutiny. Firms operating in dual-use sectors may have increased incentives to challenge regulatory actions, though this litigation pressure may also prompt courts to provide clearer interpretive guidance than agency rulemaking alone would have produced.

Building on our previous research (Reader and Atanda, 2025), we identified four anticipated consequences of *Loper Bright* on regulatory actions. First, agencies may experience constrained administrative authority as courts assert greater control over statutory interpretation. Second, agency rulemaking may be delayed, especially in time-sensitive biosecurity contexts, as agencies invest additional time in “Loper-proofing” the language they use to create policies. Third, the regulatory landscape may become complex and fragmented, since the courts are now required to “use every tool at their disposal to determine the best reading of the statute and resolve the ambiguity,” rather than defer to agency expertise. Finally, both the Legislature and Judiciary will face increased institutional burdens, requiring intense technical knowledge, expanded staffing or reliance on technical subject matter experts. It is worth noting, that this shift may also reduce certain burdens on agencies themselves, those operating in legally ambiguous areas may spend less time in litigation, even as their overall interpretive authority is constrained.

This post-*Loper Bright* environment poses distinctive challenges for biosecurity governance, particularly in capabilities involving dual-use technologies which have tremendous potential for utility and capacity to cause harm. Synthetic nucleic acid synthesis enables the rapid construction of DNA and RNA sequences for research, therapeutics, vaccine development, and industrial biotechnology. At the same time, the same capabilities that accelerate vaccine development can be misused to recreate pathogenic viruses or engineer novel biological threats. Oversight mechanisms including screening requirements for gene synthesis providers, export controls, biosafety regulations, and FDA review of nucleic acid–based therapeutics, often depend on agency interpretations of broadly worded statutes enacted decades before contemporary synthetic biology emerged.

*Loper Bright* raises a foundational institutional question: in domains where subject-matter expertise is essential to evaluating risk, innovation, and societal tradeoffs, how should interpretive authority be allocated between courts and expert agencies? The answer will profoundly shape the future of U.S. biosecurity governance.

Against this backdrop, this paper analyzes the landscape of U.S. federal regulations around biosecurity and synthetic nucleic acid technologies in the post-*Loper Bright* era. We empirically examine regulatory trends and temporal patterns to answer the following questions:

1. Which federal departments, agencies or offices are issuing synthesis-relevant regulatory actions, and how has that distribution shifted over time?
2. Do domains with clearer (high clarity) statutory authority show greater resilience to the *Loper Bright* decision?
3. Do temporal patterns reveal emerging gaps, fragmentation, or shifts in oversight–particularly around major governance milestones such as *Loper Bright* and related administrative law developments?

By mapping regulatory activity across federal departments, agencies or offices over time, we seek to characterize the post-*Loper Bright* administrative landscape. We anticipate an uneven distribution of oversight, with heightened activity among federal departments or agencies whose statutory mandates directly intersect with biosafety, biosecurity, or biotechnology governance. Agencies or offices operating under well-defined legislative authority demonstrated relatively stable regulatory patterns, whereas those navigating emerging or contested governance spaces may display more variable or episodic activity, particularly in proximity to major policy or judicial developments. Emerging domains in life sciences such as sequence screening, customer verification, and environmental release may show fragmented regulatory attention, reflecting statutory ambiguity, overlapping jurisdiction, or institutional capacity constraints.

Through this structured analysis, we aim to contribute to ongoing debates about the appropriate allocation of authority between courts and expert federal departments, agencies and offices in the governance of dual-use technologies.

## 2 Materials and methods

We conducted a structured landscape analysis using a surveillance-style analytic framework to characterize federal oversight of synthetic nucleic acid technologies before and after the Supreme Court’s decision in *Loper Bright Enterprises v. Raimondo* (28 June 2024), which overruled *Chevron* deference. This observational study compared regulatory activity across multiple time periods using pre-post comparisons, rolling-window analysis, and administration-period partitioning to assess changes in federal biosecurity governance. We extracted regulatory documents from Regulations.gov, the U.S. federal government’s official repository for regulatory materials. Our search included three document types: Final Rules, Proposed Rules, and Notices. These represent the spectrum of formal regulatory activity, from binding regulations to public communications and guidance.

### 2.1 Search strategy

We identified a set of search terms (Table 1) that capture essential components of the synthetic nucleic acid ecosystem, including synthetic nucleic acid, gene synthesis, nucleic acid screening, and other biosecurity governance-linked terminology (biosecurity, biotechnology and bioeconomy).

**TABLE 1:** Search terms and regulatory domains.

Search term	Regulatory domain captured
Biosecurity	Broad policy and security-focused regulatory actions
Biotechnology	Actions under the 1986 Coordinated Framework and general biotechnology oversight
Bioeconomy	Economic applications including biofuels and biomanufacturing
Synthetic nucleic acid	Emerging governance efforts including the 2024 OSTP screening framework
Gene synthesis	Dual-use oversight of nucleic acid synthesis services and providers
Nucleic acid screening	Diagnostic and blood safety regulations under FDA authority

These terms were applied to publicly accessible federal regulatory records on the regulations.gov database to identify potentially relevant policies. Our search tracked regulatory activity in 26 federal agencies or offices across 14 federal departments, collectively federal DAOs (departments, agencies or offices) going forward–see Supplementary Table S1 for complete list.

To enable analyses requiring document-level posting dates–the rolling-window placebo, the administration transition split, and the matched certiorari-period baseline–we constructed a parallel dataset using the Federal Register API, which provides machine-readable access to individual document metadata including posting dates, agency identifiers, and document types. We applied the same keyword terms and document type restrictions as the primary protocol across January 2013 through May 2025. For analyses using this parallel dataset, Federal Register API counts were used for new analytical windows, and primary dataset counts were used for the post-Loper Bright window. We refer to this as the hybrid approach. The two sources return directionally consistent results for narrow keywords (gene synthesis, synthetic nucleic acid, biosecurity) and diverge for broader keywords (biotechnology); where they diverge, both results are reported transparently.

To validate keyword construct validity, three independent raters (D.I., K.S., S.R.) each classified a random sample of 15 documents per keyword (45 documents total, covering biotechnology, biosecurity, and gene synthesis) as On-topic, Tangential, or Off-topic. Inter-rater agreement was assessed using Fleiss’ kappa. Overall kappa was 0.473 (moderate agreement). Per-keyword kappa was 0.531 for biosecurity (moderate), 0.639 for gene synthesis (substantial), and 0.100 for biotechnology (slight). By majority vote, 67% of biosecurity documents were on-topic (13% tangential, 20% off-topic), 33% for biotechnology, and 33% for gene synthesis; 47% of gene synthesis documents were classified as off-topic, reflecting incidental keyword matches. Results are reported in Supplementary Table S3.

### 2.2 Analytical framework and statistical approach

We employed five (Skidmore v. Swift and Co, 1944) complementary analytical approaches:

1. Administration Effect Analysis: We compared annualized regulatory activity between the Trump (2017–2020) and Biden (2021–2024) administrations to assess whether observed changes could be attributed to administration policy preferences rather than *Loper Bright.*
2. Loper Bright Effect Analysis: We compared equal 12-month windows immediately before (June 2023–May 2024) and after (June 2024–May 2025) the Loper Bright decision. We note that the post-decision window spans the administration transition of January 2025; this is examined directly in the administration transition analysis below and in Table 6.
3. Anticipatory Effects Analysis: We compared annualized activity across pre-certiorari, certiorari period, and post-decision periods to assess whether federal DAO’s began adjusting regulatory behavior after the Supreme Court signaled it would reconsider *Chevron* deference, but before the decision was issued.
4. Administration Transition Analysis: We partitioned the post-Loper Bright window at 20 January 2025, to assess whether the observed decline is concentrated in the Biden-administration tail (28 June 2024 to 19 January 2025) or the Trump II segment (January 20 to 31 May 2025), addressing the possibility that executive policy priorities contributed to the observed decline.
5. Rolling-Window Placebo Analysis: We computed year-over-year percent changes across 112 consecutive rolling 12-month windows from January 2013 through May 2023 to characterize baseline variability in regulatory output. Observed post-Loper Bright changes were placed in this null distribution and their empirical percentiles reported.

To enable the administration effect and Loper Bright effect analyses described above, we collected data across four time periods as shown in Table 2.

**TABLE 2:** Analytic time periods.

Period	Date range	Duration
Trump Administration	1 January 2017 – 31 December 2020	4 years
Biden Administration	1 January 2021 – 31 December 2024	4 years
Pre-Loper Bright 12-month	1 June 2023 – 31 May 2024	1 year
Post-Loper Bright 12-month	1 June 2024 – 31 May 2025	1 year
Biden-administration tail	28 June 2024 – 19 January 2025	206 days
Trump II segment	20 Jane 2025 – 31 May 2025	132 days
Matched pre-certiorari baseline	30 July 2023 – 12 January 2024	167 days
Certiorari period	13 January 2024 – 27 June 2024	167 days

Additionally, we constructed a three-period framework for the anticipatory effects analysis as shown in Table 3.

**TABLE 3:** Three-period framework for certiorari analysis.

Period	Date range*	Duration
Matched pre-certiorari baseline	30 Jul 2023 – 12 Jan 2024*	167 days
Certiorari Period	13 Jan 2024 – 27 Jun 2024	167 days
Post-Decision	28 Jun 2024 – 31 May 2025	338 days

* The matched pre-certiorari baseline (30 July 2023 to 12 January 2024) is equal in length to the certiorari period (167 days) and immediately precedes it, enabling direct comparison across equal windows within the same regulatory environment. Primary dataset counts are used for the post-decision window; Federal Register API counts are used for the matched pre-certiorari and certiorari windows.

For all analyses, we calculated percent change in regulatory issuance counts between comparison periods. For periods of unequal duration, we annualized counts before comparison. This approach allowed us to examine regulatory activity relative to key governance events, including administration changes, synthesis-screening policy developments, and the *Loper Bright* decision. We report descriptive statistics including total documents, annualized rates, percent change, and agency-level distributions. We additionally report exact Poisson rate-ratio tests, 95% confidence intervals via the F-distribution method (Sahai and Khurshid 1993), and single-document reclassification sensitivity analyses for all keyword-level comparisons.

We assessed the statutory basis for each keyword domain by reviewing primary authorizing statutes and rating statutory clarity as high, medium, and low. High clarity reflects explicit statutory mandates with clear jurisdictional boundaries. Medium clarity reflects existing statutory authority that requires interpretation or involves shared jurisdiction. Low clarity reflects the absence of a dedicated statute, relying instead on guidance, coordination authority, or broad interpretation of general mandates. This assessment enabled testing of the hypothesis that domains with clearer statutory authority (high statutory clarity) show greater resilience to the Loper Bright decision.

This study identifies patterns in regulatory activity over time but cannot definitively show that *Loper Bright* caused these changes. Other factors may have contributed. However, by comparing multiple time periods and using the analytical approaches described above, we can assess whether the patterns are consistent with a *Loper Bright* effect, noting that the concurrent administration transition in January 2025 represents a confound that cannot be fully disentangled from a judicial effect.

## 3 Results

Overall, our search identified 141 biosecurity-relevant regulatory issuances between June 2023 and May 2025, representing 0.3% of the 45,700 total federal regulatory issuances during this period. In the 12 months following *Loper Bright*, biosecurity-relevant activity declined 21.5% (79–62 issuances). This overall decline does not reach conventional statistical significance (RR = 0.785, 95% CI [0.551, 1.114], p = 0.178). This decline occurred against a backdrop of increasing overall federal regulatory output, which rose 2.0% during the same period (22,624 to 23,076 total issuances). The decline also contrasts with longer-term trends: comparing full presidential terms, biosecurity regulatory activity increased 12.2% from the Trump administration (288 total; 72 annually) to the Biden administration (323 total; 81 annually), noting that the Biden administration period used here (2021–2024) predates the post-Loper Bright window and does not include the Trump II segment. Together, these comparisons indicate the decline is specific to the biosecurity domain and temporally coincident with the post-Loper Bright period, though the concurrent administration transition in January 2025 represents a confound discussed further below.

### 3.1 Keyword-level analysis

Regulatory changes varied substantially across keyword domains as shown in Table 4.

**TABLE 4:** Regulatory activity change by keyword domain.

Keyword	Pre-Loper 12 months	Post-Loper 12 months	Change	% Change	Rate ratio	95% CI	p- value
Gene synthesis	8	1	−7	−87.5%	0.125	[0.003, 0.930]	0.039*
Synthetic nucleic acid	6	3	−3	−50.0%	0.500	[0.082, 2.344]	0.508
Biotechnology	39	28	−11	−28.2%	0.718	[0.431, 1.197]	0.222
Biosecurity	16	13	−3	−18.8%	0.812	[0.362, 1.797]	0.711
Nucleic acid screening	8	11	+3	+37.5%	1.375	[0.502, 3.937]	0.648
Bioeconomy	2	6	+4	+200.0%	3.000	[0.542, 30.390]	0.289
Total	79	62	−17	−21.5%	0.785	[0.551, 1.114]	0.178

* p < 0.05. Two-sided exact conditional Poisson test. 95% CIs via F-distribution method (Sahai and Khurshid 1993). Rate ratio = post/pre count ratio across equal 12-month exposure windows. Pre-Loper: 1 June 2023 – 31 May 2024. Post-Loper: 1 June 2024 – 31 May 2025. The gene synthesis p-value (0.039) is computed on raw keyword-search counts; validation (Supplementary Table S3) indicates a 47% off-topic rate for this keyword, and under validation-adjusted counts the result does not reach conventional significance (p = 0.125 to 0.250 across noise models). The directional finding and the rolling-window result (Figure 2) are robust to this adjustment.

**FIGURE 1:**
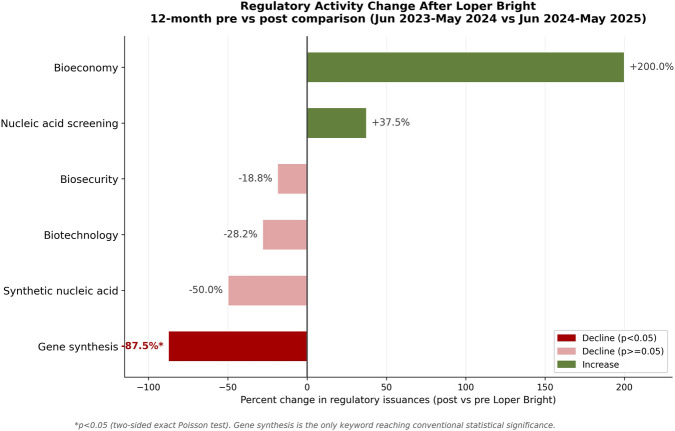
Regulatory activity change after *Loper Bright*.

As shown in Figure 1, the most severe decline occurred in gene synthesis (−87.5%, RR = 0.125, 95% CI [0.003, 0.930], p = 0.039), a domain with no dedicated federal statute, and the only keyword to reach conventional statistical significance on raw counts, though as detailed below this significance does not survive adjustment for keyword validation. The remaining individual keywords and the overall 21.5% total decline did not reach statistical significance, indicating the broader pattern is directionally consistent but not individually significant at the keyword level. Statistical tests, confidence intervals, and single-document sensitivity analyses are reported in Table 4. Synthetic nucleic acid (−50.0%) also showed substantial decline. Biotechnology (−28.2%) showed a decline under the primary dataset, though keyword validation identified a high tangential rate for this domain and the finding should be interpreted cautiously. In contrast, nucleic acid screening (+37.5%) and bioeconomy (+200.0%) showed increased activity. Both domains have clear statutory authority under the FDCA/PHSA and Clean Air Act respectively.

**FIGURE 2:**
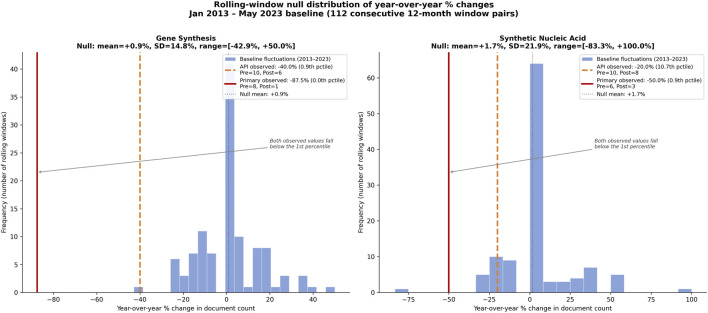
Rolling-window null distribution of regulatory output, January 2013 through May 2023.

The statistical significance of the gene synthesis decline is sensitive to keyword validation. The Poisson test above is computed on raw keyword-search counts (Pre = 8, Post = 1), which the validation exercise (Supplementary Table S3) indicates include approximately 47% off-topic documents. Adjusting for this off-topic rate under a proportional-noise model, scaling each count by the 33% on-topic rate, yields approximate counts of Pre = 3, Post = 0 (p = 0.250). Under a constant-background model, in which a fixed number of false positives appears in each window, the estimated false-positive floor of approximately 3.7 documents per window exceeds the raw post-period count of 1, driving the validated post count to zero and yielding approximately Pre = 4, Post = 0 (p = 0.125). Under neither model does the result reach conventional significance, a consequence of the small absolute counts. The direction of the change is preserved under both models, as the validated post-period count falls to zero or near-zero in each case, indicating a substantial decline regardless of the adjustment applied. The rolling-window placebo result (Figure 2) provides further support: because it is based on percent change between windows, off-topic noise that is proportional cancels in the ratio, leaving the decline’s position in the null distribution unchanged, while under a constant-background model the proportional decline becomes more extreme. The rolling-window finding is invariant under the proportional-noise model and strengthens under the constant-background model, and does not depend on the absolute counts, which is why we rest the gene synthesis interpretation on it rather than on the raw-count significance.

### 3.2 Administration effect vs. Loper-Bright effect

To distinguish administration policy effects from *Loper Bright* effects, we compared both factors for each keyword (Table 5). Administration Effect reflects the percent change in annualized activity between the full Trump term (2017–2020) and full Biden term (2021–2024). *Loper Bright* Effect reflects the percent change between the 12 months before and 12 months after the decision.

**TABLE 5:** Decomposition of administration and *Loper Bright* effects.

Keyword	Admin effect (trump 2017–20 vs. biden 2021–24)	Loper effect (12 months Pre vs. post decision)	Pattern
Biosecurity	+37.0%	−18.8%	Growth reversed after Loper Bright
Biotechnology	+1.1%	−28.2%	Growth reversed after Loper Bright
Synthetic nucleic acid	+200.0%	−50.0%	Growth reversed after Loper Bright
Gene synthesis	−40.0%	−87.5%	Continued decline
Nucleic acid screening	+33.3%	+37.5%	Sustained increase
Bioeconomy	+500.0%	+200.0%	Sustained increase
Total	+12.2%	−21.5%	Growth reversed after Loper Bright

**FIGURE 3:**
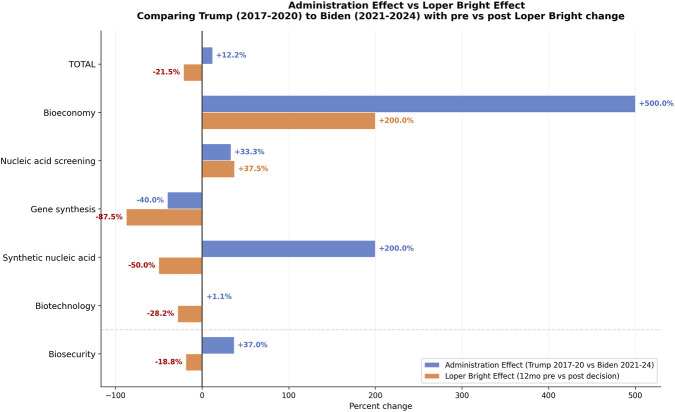
Administration effect vs. *Loper Bright* effect.

**FIGURE 4:**
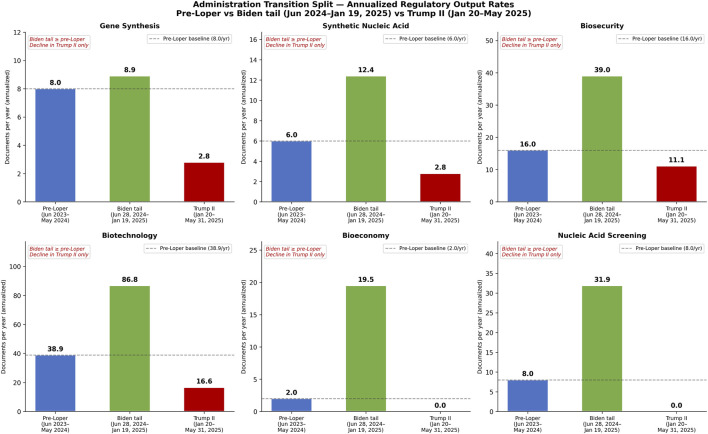
Annualized regulatory output rates by administration period.

The predominant pattern was increased activity during the Biden administration but declined after *Loper Bright* as seen in Figure 3, though administration transition analysis partitioned at 20 January 2025 shows that post-Loper Bright rates during the Biden tail were at or above the pre-Loper Bright baseline for all keywords, with the decline concentrated in the Trump II segment (see Table 6; Figure 4). This pattern appeared for biosecurity, biotechnology, synthetic nucleic acid, and the overall total. Gene synthesis showed decline during both periods. Nucleic acid screening and bioeconomy showed increases during both periods.

**TABLE 6:** Administration transition split-annualized regulatory output.

Keyword	Pre-loper n	Pre-loper rate/yr	Biden tail n	Biden tail rate/yr	Trump II n	Trump II rate/yr	Decline concentrated in
Gene Synthesis	8	8.0	5	8.9	1	2.8	Trump II
Synthetic nucleic acid	6	6.0	7	12.4	1	2.8	Trump II
Biosecurity	16	16.0	22	39.0	4	11.1	Trump II
Biotechnology	39	38.9	49	86.8	6	16.6	Trump II
Bioeconomy	2	2.0	11	19.5	0	0.0	Trump II
Nucleic acid screening	8	8.0	18	31.9	0	0.0	Trump II

Pre-Loper = 1 June 2023 – 31 May 2024 (366 days); primary dataset counts. Biden tail = 28 June 2024 – 19 January 2025 (206 days); Federal Register API counts. Trump II = 20 January 2025 – 31 May 2025 (132 days); Federal Register API counts. Rate/yr = (n/days) x 365. Biden tail rates are at or above the pre-Loper baseline for all six keywords. Decline is concentrated in the Trump II segment across all keywords.

### 3.3 Anticipatory effects

Using a matched 167-day pre-certiorari baseline (30 July 2023 to 12 January 2024), gene synthesis output rose during the certiorari period (8.7/yr to 13.1/yr, +50%) before declining sharply post-decision (1.1/yr under primary dataset, 6.5/yr under Federal Register API). Results are reported in Table 7.

**TABLE 7:** Matched certiorari-period baseline analysis.

Keyword	Matched pre n	Matched pre rate/yr	Cert n	Cert rate/yr	Cert %Δ vs. pre	Post n (API)	Post rate/yr (API)	Post%Δ vs. pre (API)	Post n (Primary)
Gene synthesis	4	8.7	6	13.1	+50.0%	6	6.5	−25.9%	1
Synthetic nucleic acid	6	13.1	3	6.6	−50.0%	8	8.6	−34.1%	3
Biosecurity	15	32.8	6	13.1	−60.0%	26	28.1	−14.4%	13
Biotechnology	23	50.3	19	41.5	−17.4%	55	59.4	+18.2%	28
Bioeconomy	1	2.2	2	4.4	+100.0%	11	11.9	+443.5%	6
Nucleic acid screening	5	10.9	3	6.6	−40.0%	18	19.4	+77.9%	11

Matched pre-certiorari baseline = 30 July 2023 – 12 January 2024 (167 days); Federal Register API., Certiorari period = 13 January 2024 – 27 June 2024 (167 days); Federal Register API. Post-decision = 28 June 2024 – 31 May 2025 (338 days). Post API = Federal Register API counts. Post Primary = original Regulations.gov dataset. Rate/yr = (n/days) x 365. %Δ = percent change vs. matched pre-certiorari baseline. Both post sources agree on direction of decline for gene synthesis, synthetic nucleic acid, and biosecurity.

When compared against the original 11-year pre-certiorari baseline, annualized activity across all keywords appeared to decline approximately 60% during the certiorari period, though this comparison involves unequal windows spanning multiple administrations and policy environments.

**FIGURE 5:**
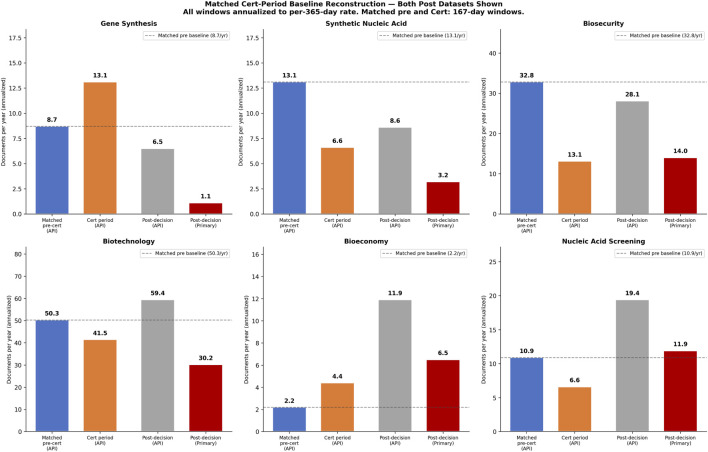
Matched certiorari-period baseline analysis.

Results varied across keywords (Figure 5). Biotechnology showed a large decline during the certiorari period under the matched baseline, though the Federal Register API and primary dataset diverge for this keyword and the finding should be interpreted cautiously.

To assess whether the post-Loper Bright decline is unusual relative to normal year-over-year variation, we constructed a rolling-window null distribution using 112 consecutive 12-month window pairs from January 2013 through May 2023. The observed gene synthesis decline (−87.5% under primary dataset, −40% under Federal Register API) falls at or below the 0^th^ percentile under the primary dataset–below all 112 historical window pairs–and at the 0.9^th^ percentile under the Federal Register API dataset, indicating the primary dataset result is more extreme than every historical year-over-year fluctuation in the baseline period, and the API result more extreme than 99% of them. The most extreme historical fluctuation for gene synthesis was −42.9%, less than half the observed magnitude. Synthetic nucleic acid (−50.0% primary) falls at the 0.9^th^ percentile under primary counts and the 10.7^th^ percentile under API counts. Results are reported in new Figure 2.

### 3.4 Statutory clarity and regulatory resilience

Table 8 compares each keyword’s statutory clarity with its observed regulatory change. Percent change reflects the difference in regulatory issuances between the 12 months before *Loper Bright* (June 2023–May 2024) and 12 months after (June 2024–May 2025).

**TABLE 8:** Regulatory changes correlated with statutory clarity.

Keyword	Primary statutory basis	Clarity	% Change
Gene synthesis	None; agencies relied on broad interpretation	Low	−87.5%
Synthetic nucleic acid	2024 OSTP screening framework (executive guidance; not codified in statute)	Low	−50.0%
Biotechnology	1986 Coordinated Framework (outdated, fragmented)	Low	−28.2%
Biosecurity	Multiple statutes (PHSA including Select Agent provisions, PPA, AHPA)	Medium	−18.8%
Nucleic acid screening	FDCA, PHSA (diagnostics and blood safety)*	High	+37.5%
Bioeconomy	Clean Air Act (explicit biofuels mandate)	High	+200.0%

* Our search term “nucleic acid screening” primarily captures FDA-regulated diagnostic and blood safety activities, which have clear statutory authority under the FDCA and PHSA. This is distinct from nucleic acid synthesis order screening—the process of vetting DNA synthesis orders for biosecurity concerns—which lacks dedicated statutory authority and is the subject of pending legislation.

Keywords with low statutory clarity showed the largest declines, while keywords with high statutory clarity showed increases, consistent with the hypothesis that statutory clarity correlates with regulatory resilience in the post-Loper Bright environment.

Two domains that share similar terminology warrant distinction. Nucleic acid screening for diagnostics and blood safety falls under clear FDA authority (FDCA, PHSA), and our search results primarily reflect regulatory activity in this domain. In contrast, screening of nucleic acid synthesis orders for biosecurity purposes lacks clear statutory authority. Pending legislative efforts aim to address this gap. The high-clarity rating for “nucleic acid screening” in this analysis reflects the diagnostic domain captured by our search, not synthesis order screening.

### 3.5 Agency-level findings

Among the 26 federal DAOs tracked, regulatory activity was concentrated in a subset with direct biosecurity mandates. APHIS, EPA, FDA, and CDC collectively accounted for the majority of activity in both periods (57% pre-Loper Bright, 52% post-*Loper Bright*). Note that the post-Loper Bright period includes both the Biden-administration tail (28 June 2024 to 19 January 2025) and the Trump II segment (January 20 to 31 May 2025); agency-level counts are not disaggregated by administration here but the overall decline is discussed in Section 3.2.

Post-Loper Bright, the top three federal DAOs shifted from EPA (Jeffery, 2025), APHIS (Pennsylvania State University and Office for Research Protections, 2025), and FDA (Bailey and Maltzman, 2011) to APHIS (United States Executive Office of the President, 2025), EPA (Carrubba et al., 2008), and USDA (Brannon, 2023), noting APHIS is an agency under USDA (department). DOJ activity increased from 1 to 5 issuances, an intriguing but small-sample observation that may suggest a shift toward enforcement of existing authorities, though this warrants further examination with larger samples before drawing firm conclusion. See Supplementary Table S2 for agency-level regulatory activity, pre vs. post *Loper Bright*.

### 3.6 Cross-validation of findings

Findings were consistent across most analytical approaches, with one informative exception noted below:

1. Administration effect analysis showed biosecurity activity increased 12.2% from the Trump to Biden administration, suggesting the decline is not solely attributable to Biden-era policy preferences. However, administration transition analysis partitioned at 20 January 2025 shows the post-Loper Bright decline is concentrated in the Trump II segment, indicating concurrent executive policy priorities cannot be ruled out as a contributing factor.
2. *Loper Bright* effect analysis quantified the post-decision decline at 21.5% overall, with gene synthesis showing the steepest drop at 87.5%, though the conventional statistical significance of this result does not survive keyword validation adjustment (see Section 3.1).
3. Matched certiorari-period analysis using an equal 167-day baseline showed gene synthesis output rose during the certiorari period before declining post-decision. This is the exception to the consistency noted above: it refines the timing of the decline, indicating the decline follows rather than precedes the judicial decision for this keyword, rather than contradicting the overall post-Loper pattern.
4. Statutory clarity assessment showed a pattern consistent with the hypothesis that legal ambiguity correlates with regulatory decline, with low-clarity keywords showing the largest declines.

The directional consistency across analytical approaches is consistent with a Loper Bright effect. The concurrent administration transition in January 2025 and the small absolute counts for several keywords mean the design cannot fully disentangle judicial and executive drivers.

## 4 Discussion

The doctrine of stare decisis shapes regulatory stability by establishing predictable legal frameworks on which agencies rely when developing rules (Brannon, 2023). The Loper Bright ruling departed from this principle, and this paper has examined its consequences for biosecurity governance in one specialized domain.

We have examined how the U.S. federal regulatory landscape is shifting in the face of political preferences (major policy changes), judicial decisions overturning precedent and shifting statutory clarity. Our analysis provides insight into temporal patterns and regulatory trends for federal DAOs issuing nucleic acid synthesis relevant regulations, and the gaps and shifts in oversight that result. We identified an uneven distribution of oversight with high activity in some federal departments or agencies that have biosecurity-relevant mandates. These federal DAOs with well-defined mandates showed stable regulatory patterns, while those operating in emerging areas of work showed variable patterns.

### 4.1 Disentangling political preference from judicial constraint

The observed 21.5% decline in biosecurity-relevant regulatory activity following the *Loper Bright* decision warrants careful interpretation given the concurrent administration transition in January 2025. While overall regulatory activity actually increased by 12.2% when comparing the Trump and Biden administrations, the “Admin ↑, *Loper* ↓” pattern found in key domains like biosecurity and synthetic nucleic acids suggests that these highly technical areas may be uniquely sensitive to the shifting judicial landscape, potentially tempering executive priorities. While Loper Bright only strictly alters the standard of judicial review rather than forbidding agency rulemaking, the heightened risk of litigation may prompt agencies to proactively self-censor or withhold novel regulations in legally vulnerable domains.

When the post-Loper Bright window is partitioned at 20 January 2025, annualized output rates during the Biden-administration tail (28 June 2024 to 19 January 2025) were at or above the pre-Loper Bright baseline for every keyword–gene synthesis: 8.9/yr versus baseline 8.0/yr; biosecurity: 39.0/yr versus baseline 16.0/yr. Rates collapsed in the Trump II segment (January 20 to 31 May 2025) across all keywords–gene synthesis: 2.8/yr; bioeconomy and nucleic acid screening: 0/yr. Both a Loper Bright judicial effect and executive policy priorities under the new administration may be operating concurrently; the present design cannot attribute the observed decline to either factor in isolation. Results are reported in Table 6 and Figure 4.

Prevailing evidence suggests political preferences of executive and legislative bodies impact judicial decisions in systematic ways. Carrubba et al. (2008) found this to be true in their examination of judicial behavior under political constraints in the European Court of Justice, noting the outsized role of threats of noncompliance and legislative override. Similarly, political and legal explanations weave together in a complex knot and separating these influences is key to understanding judicial decisions in the U.S. too (Bailey and Maltzman, 2011). Here, Gardner et al. (2023) show political preferences or constraints are most effective when the legislature is aligned with the executive branch which is less inclined to veto legislation, and this pattern may partially account for the increased overall regulatory activity we observed during the Biden administration.

While judicial decision-making is often seen as a neutral interpretation of the law, in reality, it is a multifaceted process shaped by a combination of institutional limitations, political dynamics, and personal perspectives all culminating to inform governance and legal outcomes.

### 4.2 Statutory clarity and regulatory resilience

Regulatory resilience in the post-*Chevron* era appears strongly associated with the explicitness of the underlying authorizing statutes. Our findings show a pattern consistent with a correlation between legal fragility and statutory ambiguity: domains relying on broad interpretations or non-binding guidance, such as gene synthesis (−87.5%) and synthetic nucleic acids (−50.0%), showed the most significant contractions. Conversely, domains with clear, dedicated mandates like the bioeconomy (+200%) and nucleic acid screening (+37.5%) showed increased activity, suggesting that specific legislative language is strongly associated with stable biosecurity governance in the post-Loper Bright environment.

Clear statutory language reduces ambiguity in agency rulemaking, making it easier for courts to interpret and enforce regulations. This precision supports more consistent judicial review, which in turn strengthens the predictability and legitimacy of regulatory outcomes, with courts less likely to strike down rules on grounds of overreach or misinterpretation (Bull and Ellig, 2019). A case study by Harker and Reader (2022) using a series of semi-structured elite interviews with senior members of the energy ‘regulatory community’ showed that the increased complexity posed by a proliferation of statutory objectives has obscured the appropriate contours and rationales of regulation for that field. Our findings are consistent with this dynamic in the gene synthesis and synthetic nucleic acid domains. Driving this is the state of transition of the 2024 Framework for Nucleic Acid Synthesis Screening (National Science and Technology Council, 2024) due to a major executive policy shift in mid-2025 (United States Executive Office of the President, 2025) where implementation was paused after only 1 month (Pennsylvania State University and Office for Research Protections, 2025). Although the 90-day imposed deadline has passed for the release of an “updated Framework”, institutions still await new specific guidance. While technically defunct as a result of the May 2025 Executive Order (Improving the Safety and Security of Biological Research), the 2024 Framework remains the current “draft” baseline until the formal 2026-ready version is published.

### 4.3 Temporal patterns and the certiorari period

Analysis of temporal patterns across the certiorari timeline reveals a mixed picture. Under a matched 167-day baseline immediately preceding certiorari grant, gene synthesis output rose during the certiorari period (8.7/yr to 13.1/yr) before declining sharply post-decision (1.1/yr under primary dataset). This pattern is more consistent with a post-decision effect than an anticipatory one. Under the original 11-year pre-certiorari baseline, annualized activity across all keywords appeared to decline approximately 60% during the certiorari period, though this comparison spans multiple administrations and policy environments. Whether the observed patterns reflect anticipatory regulatory adjustment requires qualitative corroboration, such as agency interviews or internal documentation, that the present observational design does not provide.

The temporal patterns we report are consistent with, but do not prove, a chilling effect. Future research using qualitative methods (agency interviews, internal memos, notice-and-comment records) would be needed to establish whether legal uncertainty was actively shaping agency behavior during the certiorari period and to what degree the observed post-decision decline reflects judicial constraint, executive policy priorities, or both operating concurrently.

### 4.4 Increasing governance concentration and regulatory gaps

As the legal landscape shifts, biosecurity governance appears increasingly concentrated among a few departments or agencies with the most robust statutory foundations, with “novel” domains showing the greatest vulnerability. The reduced activity of departments or agencies operating under ambiguous mandates has resulted in oversight increasingly concentrated among a shrinking subset of regulators like the USDA (APHIS), EPA, and FDA. This concentration creates significant regulatory gaps, particularly for technically specific terms like gene synthesis, where the absence of a dedicated statute is associated with a substantial withdrawal of federal activity, potentially leaving emerging biosecurity threats undermonitored. Regulatory gaps, vulnerabilities, and possible legislative fixes are provided in Table 9.

**TABLE 9:** Regulatory gaps, vulnerabilities and possible legislative fixes.

Regulatory gap	Identified vulnerability	Possible legislative fix
Gene synthesis	87.5% decline in activity; no dedicated statute	Enact the “Synthetic Gene Sales Act” to mandate screening and provide explicit Commerce Dept. authority
Synthetic nucleic acids	50% decline; high reliance on voluntary OSTP frameworks	Codify the 2024 OSTP Screening Framework into federal law to provide a non-discretionary enforcement basis
Agency concentration	Governance relies on only 3–4 agencies	Amend the Coordinated Framework for Biotechnology to explicitly distribute technical oversight authorities

### 4.5 Importance of this work

Synthetic nucleic acid technologies are becoming increasingly accessible, lowering barriers to sequence modification, design, and synthesis. These capabilities heighten the need for coherent governance architectures that address sequence-level risks, screening standards, technology democratization, attribution, liability, and cross-border oversight. By providing a systematic, empirical characterization of federal oversight patterns across synthesis-relevant domains, complementing existing qualitative analyses of the post-Chevron regulatory landscape (Jeffery, 2025). This study identifies where governance is robust, where gaps are emerging, and where coordination or updated frameworks may be needed in a post-*Loper Bright* world, following the end of 40 years of *Chevron* deference by the Supreme Court in 2024. These findings can support policy development, inform international harmonization efforts, and strengthen technical and ethical standards for the safe and secure deployment of synthetic nucleic acid technologies.

So far, 19 U.S. states have already moved (Cavanaugh, 2026) to eliminate administrative deference through legislative or judicial means, establishing a framework where judges, rather than agency officials, serve as the final arbiters of legal meaning to ensure that individuals challenging state actions receive a truly neutral hearing.

In interpreting the trends from the data we used, several institutional and structural confounding factors must be considered alongside the shifting judicial landscape. First, the regulatory pipeline for complex technologies within the biosecurity space is characterized by protracted developmental timelines. Because federal rulemaking typically spans months or even years from initial drafting to final publication, attributing sharp shifts within a narrow temporal window directly to specific judicial milestones introduces significant analytical complexity, and this is further complicated by concurrent advances in other technologies, in this case, artificial intelligence. Second, observed regulatory pauses may reflect broader, concurrent shifts in executive priorities regarding emerging technology governance rather than a distinct judicial chilling effect, further complicated by exogenous influences of the 2024 U.S. election cycle and subsequent administration transition planning. In the absence of corroborating qualitative indicators, such as internal agency memos, tracking of shifted bureaucratic bandwidth, or structured interviews with regulatory officials, these findings should be viewed as consistent with a Loper Bright effect rather than an unconfounded causality. By acknowledging these competing explanations, we position our analysis as a baseline for understanding how heightened legal uncertainty interacts with long-term biosecurity policy trajectories.

### 4.6 Limitations

This study has several limitations. First, keyword-based searches may include false positives (documents mentioning terms but not addressing biosecurity) or false negatives (relevant documents using different terminology), such that percentage changes are sensitive to small shifts in data classification. Inter-rater validation yielded Fleiss’ kappa of 0.100 for biotechnology (slight agreement), 0.531 for biosecurity (moderate agreement) and 0.639 for gene synthesis (substantial agreement), with 47% of gene synthesis documents classified as off-topic by majority vote, reflecting incidental keyword matches. The statistical significance of the gene synthesis decline does not survive adjustment for this off-topic rate (validated-count p ranges from 0.125 to 0.250 across noise models), as the small absolute counts leave insufficient sample to detect significance once false positives are removed. The directional finding and its position in the rolling-window null distribution are robust to this adjustment, as detailed in Section 3.1. Second, we count regulatory documents without weighting by significance or scope. Third, some of the changes we report derive from very small denominators that are historically low-frequency and episodic, and conclusions may not be as robust as the percentage changes suggest. Fourth, the post-Loper Bright observation period is limited to approximately 12 months, precluding assessment of long-term effects. Fifth, the Loper Bright observation window spans the 2024 U.S. election cycle and subsequent administration transition, with a dense concentration of exogenous influences that precluded further disaggregation. Sixth, the administration transition on 20 January 2025 represents a concurrent factor that the design cannot fully disentangle from a Loper Bright effect. Seventh, AI governance regulatory output (searching “artificial intelligence,” “machine learning,” and “algorithmic”) across target agencies fell 14.6% in the post-Loper Bright window, arguing against a capacity-displacement explanation, though the agency-level sample available for this test is small (n = 5 agencies). Eighth, this observational design cannot definitively establish causation, and the combination of analytical approaches is consistent with but does not prove a Loper Bright effect. Despite these limitations, this descriptive analysis characterizes meaningful temporal patterns in the biosecurity regulatory landscape that may have utility for policymakers and scientists.

## 5 Conclusion

This study finds temporal patterns consistent with a Loper Bright effect on biosecurity-relevant regulatory activity, anchored on the gene synthesis domain: a −87.5% decline that is more extreme than 99% of historical year-over-year fluctuations in the 2013–2023 baseline period. This decline reaches conventional statistical significance on raw counts (p = 0.039) but not after adjustment for keyword validation, a consequence of small absolute counts; the finding that does not depend on absolute counts, its extremity relative to historical baseline variation, is robust. The broader 21.5% overall decline is directionally consistent but does not reach statistical significance. The concurrent administration transition in January 2025 represents a confound the observational design cannot fully disentangle–administration-period analysis shows output rates were at or above the pre-Loper Bright baseline during the Biden-administration tail, with the sharp decline concentrated in the Trump II segment. Whether the observed patterns reflect a Loper Bright judicial effect, executive policy priorities, or both operating concurrently requires qualitative investigation that the present design cannot provide.

The observed patterns suggest that biosecurity governance is becoming increasingly fragmented and concentrated within a few departments or agencies. Domains with clear statutory foundations, such as the bioeconomy and nucleic acid screening, remain resilient and even continue to grow. However, the decrease in regulatory activity by departments or agencies from dynamic areas like synthetic nucleic acid technology creates regulatory gaps that cannot be filled by executive action alone. To maintain a comprehensive national biosecurity posture, these findings suggest that legislative intervention may be required to provide the explicit statutory authority in domains currently dependent on broad interpretive mandates.

## Data Availability

The raw data supporting the conclusions of this article will be made available by the authors, without undue reservation.

## Supplementary material

Supplementary Table S1 List of 26 federal agencies and offices included in the analysis, grouped by department.

Supplementary Table S2 Agency-level regulatory activity, pre versus post Loper Bright, with direct biosecurity mandate designation.

Supplementary Table S3 Keyword construct validity, inter-rater agreement (Fleiss’ kappa) and classification results.

Supplementary Table S4 AI governance competing-driver test, aggregate counts and agency-level correlation.

SUPPLEMENTARY FIGURE S1 AI governance competing-driver test, agency-level correlation and aggregate output comparison.

SUPPLEMENTARY FIGURE S2 Statistical analysis of pre/post Loper Bright keyword counts, rate ratios with confidence intervals and single-document sensitivity.
