# Supplementary material for: Temporal patterns in biosecurity-related regulation before and after Loper Bright
Source: Front Bioeng Biotechnol. 2026 Jul 13;14:1838260. doi: 10.3389/fbioe.2026.1838260 (PMC13402510; doi:10.3389/fbioe.2026.1838260)
Supplement: Supplementary file 1 [file Table1.docx]

**Supplementary Tables**

Table S1. List of 26 federal agencies and offices included in this analysis

| **U.S. Federal Department** | **26 U.S. Federal Agencies or Offices** |
| --- | --- |
| Department of Agriculture | APHIS, AMS, FSIS, USDA (department-level) |
| Department of Health and Human Services | FDA, CDC, NIH, HHS (department-level) |
| - | EPA |
| Department of the Interior | FWS, USGS |
| Department of Homeland Security | DHS, CBP, CISA |
| Department of Commerce | BIS, NIST |
| Department of State | State |
| - | OSTP, USTR (Executive Office of the President) |
| - | NSF |
| Department of Labor | OSHA, DOL |
| Department of Energy | DOE |
| Department of Defense | DOD |
| Department of Justice | DOJ |
| - | NRC |

Table S2: Agency-level regulatory activity (pre vs post *Loper Bright*)

| **Agency** | **Department** | **Direct Biosecurity Mandate*** | **Pre-Loper 12mo** | **Post-Loper 12mo** | **Change** | **% Change** |
| --- | --- | --- | --- | --- | --- | --- |
| EPA | EPA | Yes* | 17 | 8 | 9 | -52.9% |
| APHIS | USDA | Yes* | 15 | 14 | -1 | -6.7% |
| FDA | HHS | Yes* | 9 | 5 | -4 | -44.4% |
| NSF | NSF | No | 6 | 3 | -3 | -50.0% |
| USDA | USDA | No | 4 | 7 | +3 | +75.0% |
| AMS | USDA | No | 4 | 0 | -4 | -100.0% |
| DHS | DHS | Yes* | 4 | 4 | 0 | 0.0% |
| BIS | Commerce | No | 4 | 2 | -2 | -50.0% |
| CDC | HHS | Yes* | 4 | 5 | +1 | +25.0% |
| NIST | Commerce | No | 3 | 2 | -1 | -33.3% |
| OSTP | EOP | No | 3 | 1 | -2 | -66.7% |
| FWS | DOI | No | 2 | 0 | -2 | -100.0% |
| FSIS | USDA | No | 1 | 3 | +2 | +200.0% |
| NIH | HHS | No | 1 | 0 | 1 | -100.0% |
| HHS | HHS | No | 1 | 0 | -1 | -100.0% |
| DOJ | DOJ | No | 1 | 5 | +4 | +400.0% |
| CBP | DHS | No | 0 | 1 | +1 | *New* |
| State | State | No | 0 | 2 | +2 | +200.0% |
| USGS | DOI | No | 0 | 0 | 0 | – |
| CISA | DHS | No | 0 | 0 | 0 | – |
| USTR | EOP | No | 0 | 0 | 0 | – |
| OSHA | DOL | No | 0 | 0 | 0 | – |
| DOL | DOL | No | 0 | 0 | 0 | – |
| DOE | DOE | No | 0 | 0 | 0 | – |
| DOD | DOD | No | 0 | 0 | 0 | – |
| NRC | NRC | No | 0 | 0 | 0 | – |
| TOTAL | – | ­– | 79 | 62 | -17 | -21.5% |

*Agencies with explicit statutory biosecurity mandates under the Select Agent Regulations, the Federal Food Drug and Cosmetic Act, the Plant Protection Act, or the Animal Health Protection Act.

Table S3 – inter-rater agreement and classification results

| **Keyword** | **On-topic (n, %)** | **Tangential (n, %)** | **Off-topic (n, %)** | **Fleiss' kappa** | **Agreement level** | **Interpretation** |
| --- | --- | --- | --- | --- | --- | --- |
| Biotechnology | 5 (33.3%) | 7 (46.7%) | 3 (20.0%) | 0.200 | Slight | Low kappa and low on-topic rate (33%) reflect genuine ambiguity– most retrieved documents are tangential rather than directly biosecurity-relevant. Claims anchored on biotechnology should be treated cautiously. |
| Biosecurity | 10 (66.7%) | 2 (13.3%) | 3 (20.0%) | 0.531 | Moderate | Majority of retrieved couments (67%) are on-topic. Moderate kappa suggests the keyword retrieves mostly relevant documents with some ambiguity at the biosecurity-vs-agricultural health boundary. |
| Gene Synthesis | 5 (33.3%) | 3(20.0%) | 7(46.7%) | 0.639 | Substantial | High off-topic rate (47%) confirms that unquoted keyword searches retrieves documents where gene and synthesis appear incidentally (e.g., endangered species listings, pesticides rules). Only 33% on-topic by majority vote |
| Overall (n=45) | 20(44.4%) | 12(26.7%) | 13(28.9%) | o.473 | Moderate | Pairwise Cohen's kappa: D.I.-K.S.=0.341, D.I.-S.R.=0.694, K.S.-S.R.=0.413. One 3-way disagreement adjudicated by senior author (see footnote). |

Kappa interpretation (Landis and Koch 1977): less than 0.20=Slight, 0.21–0.40=Fair, 0.41–0.60=Moderate, 0.61–0.80=Substantial, greater than 0.80=Almost perfect. Classification reported as majority vote across three independent raters (D.I., K.S., S.R.) each classifying 15 documents per keyword (45 total) as On-topic, Tangential, or Off-topic. Documents sampled randomly from the Federal Register API dataset (random seed=42).

Adjudication note: Document 28 (biosecurity keyword, FDA Food Traceability Rule) produced a three-way disagreement (D.I.=Tangential, K.S.=Off-topic, S.R.=On-topic) and was adjudicated as Off-topic by the senior author following review of the full document text. The primary subject is food supply chain recordkeeping; biosecurity does not appear as a substantive topic. Kappa values are computed from raw pre-adjudication ratings.

Table S4. AI Governance Competing-Driver Test

| Output Category | Pre (n) | Post (n) | % Change | Interpretation |
| --- | --- | --- | --- | --- |
| Biosecurity (gene synthesis + synthetnucleic acid | 17 | 13 | -23.5% | Declined post-Loper |
| AI governance (artificial intelligence + machine learning + algorithmic) | 199 | 170 | -14.6% | Also declined – no surge |

Agency-level correlation

| Agencies with data for both biosecurity and AI output | N = 5 |
| --- | --- |
| Pearson r (AI% Δ vs biosecurity % Δ) | R= +0.683 |
| p-value | P=0.203 |
| Interpretation | Positive correlation – agencies where AI output rose tended to show biosecurity output rising too, not falling. Opposite the capacity displacement theory |

Federal Register API dataset. Same 26 target agencies and document types as primary analysis. Pre = June 1, 2023 to May 31, 2024. Post = June 1, 2024 to May 31, 2025. AI governance keywords: 'artificial intelligence,' 'machine learning,' 'algorithmic.' Both biosecurity and AI governance output declined in the post-Loper window. The capacity-displacement hypothesis is not supported.
