# Supplementary figures and images for: Temporal patterns in biosecurity-related regulation before and after Loper Bright
Source: Front Bioeng Biotechnol. 2026 Jul 13;14:1838260. doi: 10.3389/fbioe.2026.1838260 (PMC13402510; doi:10.3389/fbioe.2026.1838260)

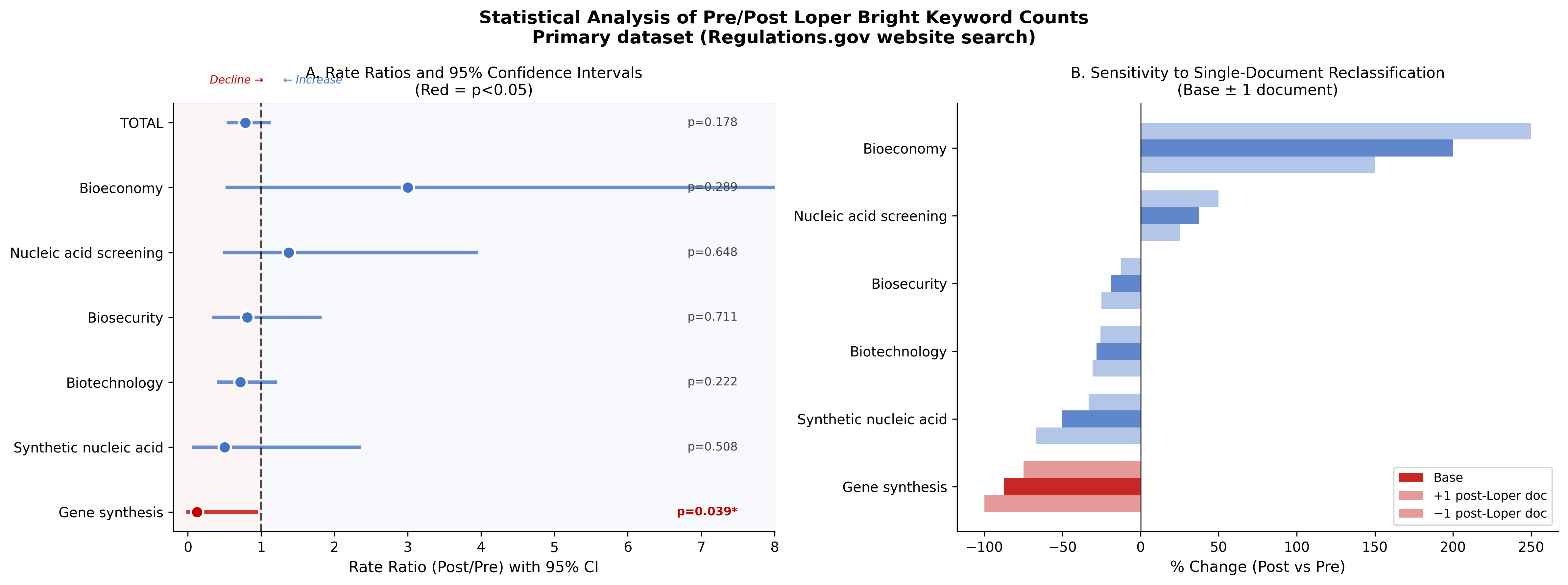

Supplement: Supplementary file 2 [file Image2.png]

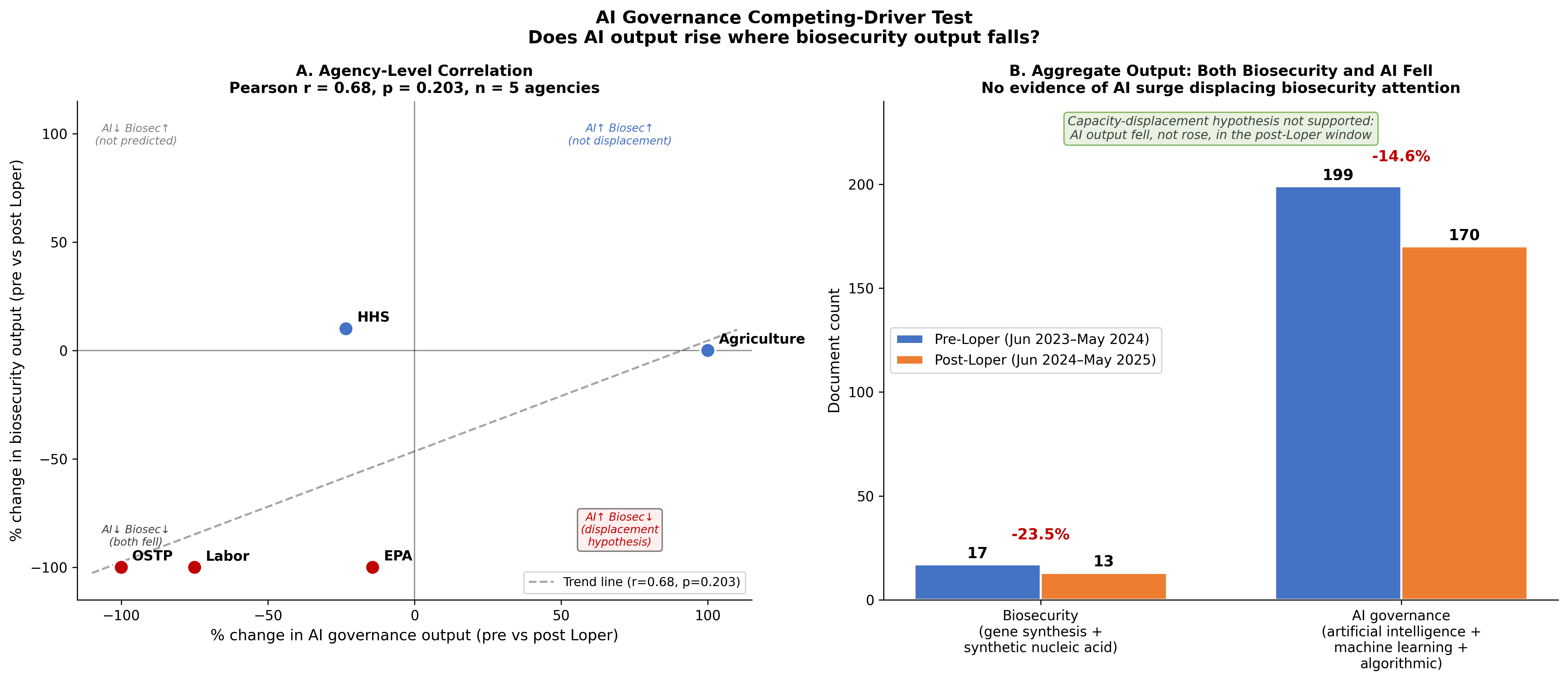

Supplement: Supplementary file 3 [file Image1.png]
